# A three-dimensional hydroxyapatite/polyacrylonitrile composite scaffold designed for bone tissue engineering†

**DOI:** 10.1039/c7ra12449j

**Published:** 2018-01-08

**Authors:** Shuyi Wu, Jieda Wang, Leiyan Zou, Lin Jin, Zhenling Wang, Yan Li

**Affiliations:** Department of Prosthodontics, Guanghua School of Stomatology, Hospital of Stomatology, Sun Yat-sen University, Guangdong Provincial Key Laboratory of Stomatology No. 56 Lingyuan Road Guangzhou 510055 P. R. China liy8@mail.sysu.edu.cn +86-20-83822807; Henan Key Laboratory of Rare Earth Functional Materials, Zhoukou Normal University 466001 P. R. China zlwang2007@hotmail.com; International Joint Research Laboratory for Biomedical Nanomaterials of Henan, Zhoukou Normal University Zhoukou 466001 P. R. China jinlin_1982@126.com

## Abstract

In recent years, various composite scaffolds based on hydroxyapatite have been developed for bone tissue engineering. However, the poor cell survival micro-environment is still the major problem limiting their practical applications in bone repairing and regeneration. In this study, we fabricated a class of fluffy and porous three-dimensional composite fibrous scaffolds consisting of hydroxyapatite and polyacrylonitrile by employing an improved electrospinning technique combined with a bio-mineralization process. The fluffy structure of the hydroxyapatite/polyacrylonitrile composite scaffold ensured the cells would enter the interior of the scaffold and achieve a three-dimensional cell culture. Bone marrow mesenchymal stem cells were seeded into the scaffolds and cultured for 21 days *in vitro* to evaluate the response of cellular morphology and biochemical activities. The results indicated that the bone marrow mesenchymal stem cells showed higher degrees of growth, osteogenic differentiation and mineralization than those cultured on the two-dimensional hydroxyapatite/polyacrylonitrile composite membranes. The obtained results strongly supported the fact that the novel three-dimensional fluffy hydroxyapatite/polyacrylonitrile composite scaffold had potential application in the field of bone tissue engineering.

## Abstract 2

A fluffy and porous (3D) HA composite fibrous scaffold was fabricated by employing an improved electrospinning technique combined with a bio-mineralization process.

## Graphical abstract

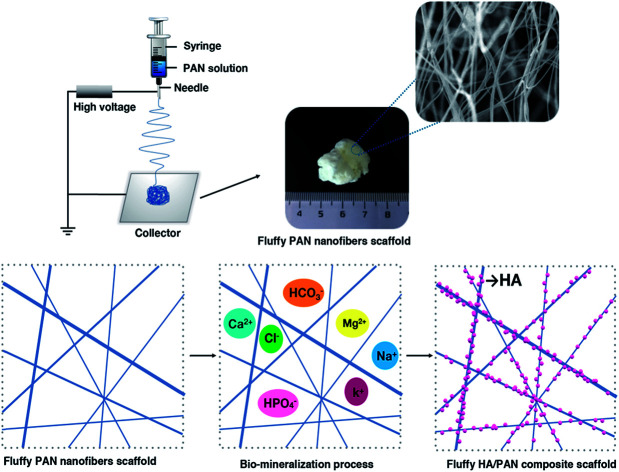

## 1. Introduction

Bone is a complicated and hierarchical structure, which is composed of hydroxyapatite (HA), primarily collagen and water.^1^ Nowadays, bone defects are a common clinical disease which significantly affects the quality of life. Various treatments are available to repair bone defects, mainly with autografts and allografts. However, there are some disadvantages hindering the application in the clinic.^2–4^ For example, the shortcomings of autografting are the limitation in donor sites and secondary damage. As for the allograft technique, the possibility of immune rejection and the spread of diseases like HIV and hepatitis are the main drawbacks.^5^ Therefore, a variety of biomaterials have been fabricated for bone repairing and regeneration in the past decades,^6–12^ such as three dimensional (3D) scaffolds,^13–16^ functional membranes^17–20^ and particles.^21–23^ In particular, 3D bone scaffolds made from polymeric materials, carrying cells or growth factors to induce the regeneration of new bone, have received increasing attention.^24,25^ Several researches have claimed that these 3D biomaterials not only possess porous structure, but also mimic the biological functions of the extracellular matrix (ECM) contributing to bone repair and regeneration.^12,26,27^

Hydroxyapatite (HA), an ideal bone substitute, which is the inorganic composition of the natural bone, with excellent biocompatibility and osteoconductivity.^28^ Nevertheless, low strength of HA limits the practical application in tissue engineering.^29^ As a result, plenty of composite scaffolds including HA/poly-lactic acid (PLA),^30,31^ HA/poly-caprolactone (PCL),^32,33^ HA/poly(lactic-*co*-glycolic) acid (PLGA),^34,35^*etc.*, have been developed for bone repairing and regeneration. The major features of ideal 3D HA based scaffolds are porous as well as high HA content. However, these current HA based bone substitutes cannot satisfy the requirements of tissue engineering due to their low HA content, which significantly limited the practical application of these HA based scaffolds. Therefore, it is necessary to develop novel 3D HA composite scaffold to meet the complicated requirements of bone tissue engineering.

In this study, we developed a novel fluffy HA based fibrous scaffold for bone tissue engineering. First, fluffy polyacrylonitrile (PAN) fibrous scaffolds were fabricated by an optimized electrospinning technique. Subsequently, the bio-mineralization process was applied to achieve HA coating on the surface of the PAN fibers. After that, the fluffy HA/PAN scaffolds with fluffy spatial fibrous structure were accomplished. The effect of the scaffolds on bone marrow mesenchymal stem cells (BMSCs) was observed over a period of 21 days culture, and assessed according to cell proliferation, morphology and osteogenic differentiation expression compared to that on two-dimensional (2D) HA/PAN composite membranes to evaluate the potential applications of the obtained HA/PAN composite scaffold in bone tissue engineering.

## 2. Experimental section

### 2.1 Materials

Poly-acrylonitrile (PAN, *M*_W_ = 110 K) was obtained from Daigang CO (Jinan, China). All other chemicals were purchased from Sigma-Aldrich and used without further purification.

### 2.2 Preparation of fluffy HA/PAN scaffolds and HA/PAN membranes

**Fig. 1:**
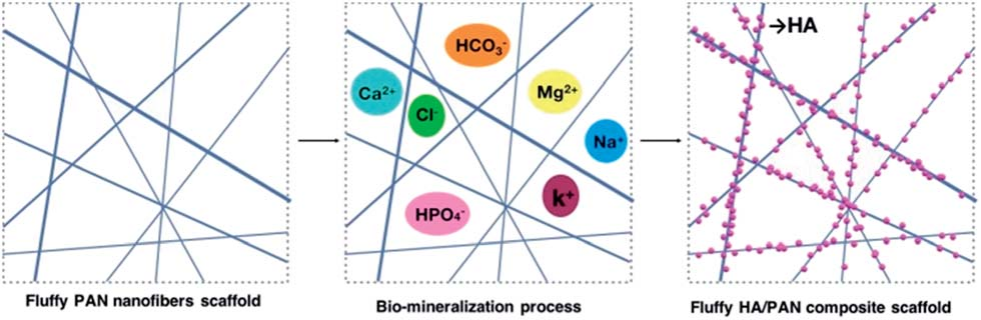
Fabrication process of 3D HA/PAN composite scaffold.

The electrospinning process was applied to fabricate the PAN nanofibers scaffolds and 2D nanofibers as previously reported (Fig. S1 and S2†).^12^ The bio-mineralization process (Fig. 1) was employed to prepare mineralized materials, and the simulated body fluid (SBF, pH = 7.2) was prepared as previously reported.^36^ Briefly, fluffy PAN scaffolds were put into a 50 ml centrifuge tube, and then, the materials were washed with SBF slightly several times after the ethanol solution was removed. Whereafter, proper amount of SBF was added into each tube to soak the materials completely and these were placed in a constant temperature shaker at 37 °C to keep the materials mineralized. The SBF was renewed every other day. At various time points, the SBF was removed and the corresponding materials were washed several times with DI water gently, then these fluffy HA/PAN composite scaffolds kept in the centrifuge tubes were dried by freeze-drying for further study. The HA/PAN composite membranes were prepared by using PAN fibrous membranes as templates in the above mineralization process.

### 2.3 Characterization of 3D HA/PAN composite scaffolds

The scanning electron microscopy (SEM) images and X-ray diffraction (XRD) of the different scaffolds were tested.

### 2.4 Isolation and culture of bone marrow mesenchymal stem cells (BMSCs)

This study was performed in strict accordance with “Laboratory Animal Science Guidance” issued by Chinese science and technology ministry for the care and use of laboratory animals and was approved by the Laboratory Animal Center of Sun Yat-Sen University (Guangzhou, China). BMSCs were isolated from 3 week-old male Sprague-Dawley rats as described previously.^37^ In short, bone marrow was aspirated from two femora of each rat, followed by centrifugation at 1000 rpm for 5 min. Then the supernatant was removed and the cell layer was re-suspended in a complete culture medium consisting of Dulbecco's modified Eagle's medium (DMEM) supplemented with 10% w/v fetal bovine serum (FBS), 100 U ml^−1^ penicillin and 100 mg ml^−1^ streptomycin (all from Gibco), and cultured in a humidified incubator (37 °C, 5% CO_2_). After 48 h, non-adherent cells were discarded. The culture medium was refreshed every 2 days until adherent cells became nearly confluent, they were expanded to passage 3–4 for further experiments.

### 2.5 Cell seeding

The obtained 3D HA/PAN composite scaffolds were cut into thin sections with a thickness of 2.0 mm and diameter of 14 mm; HA/PAN composite membranes were cut into 14 mm disks as a control, and then both were placed into 24-well plates, sterilized by soaking in a solution of 70% ethanol and 30% PBS for 12 h, followed by washing 2–3 times using PBS and complete medium. BMSCs were then seeded into/onto each 3D HA/PAN composite scaffold and HA/PAN composite membrane at a density of 1.0 × 10^4^ cells per well according to previous method.^38,39^ After 2 h cell attachment, 0.5 ml complete culture medium was added into each well. The resulting substrates/cell constructs were cultured in an incubator at 37 °C, 95% relative humidity and 5% CO_2_ partial pressure.

### 2.6 Cell cytotoxicity and proliferation

Cellular cytotoxicity and proliferation were measured using cell counting kit-8 reagent (CCK-8, Dojindo, Japan). Briefly, at the various time points including 12 h, 1 d, 3 d and 7 d, the BMSCs-seeded scaffolds and membranes were incubated in 10% CCK-8 solution at 37 °C in an incubator with 5% CO_2_ for 1 h. Afterwards, the supernatant obtained was taken to another 96-well plate and measured at 450 nm using a microplate reader.

### 2.7 Cell fluorescence detection and morphology

To evaluate cell viability on 3D HA/PAN composite scaffolds and HA/PAN composite membranes, BMSCs were seeded into/onto the substrates and cultured 3 days for cell attachment and morphology observation by fluorescence detection and SEM images.

In order to obtain fluorescence images, DAPI (Beyotime, China) was used to visualize cell nucleus, while Actin-Tracker Green (Beyotime, China) was contributed to show cytoskeleton. All cell substrates went through a series of processes containing fixation by 4% paraformaldehyde, permeabilization with 0.1% Triton-PBS and prevention of non-specific binding using 5% albumin from bovine serum (BSA) solution before stained. The fluorescence images were taken using a confocal microscope (LSM700, Zeiss).

The detailed cell morphology was assessed by SEM images. After fixed with 3% glutaraldehyde for 2 h, the BMSCs-HA/PAN composite scaffolds were kept at −20 °C for shaping and then freeze-drying. On the other hand, the BMSCs-HA/PAN composite membranes were dehydrated using gradient elution method; subsequently they were dried in the air. All the cell substrates were sputter-coated by gold and palladium before observing under the SEM.

### 2.8 *In vitro* osteogenesis study

The alkaline phosphatase (ALP) activity and calcium content were measured to assess the osteogenesis differentiation of BMSCs. Osteogenic induction medium consists of DMEM, 10% FBS, 0.1 mM dexamethasone (Sigma-Aldrich, USA), 10 mM *b*-glycerophosphate (Calbiochem, USA) and 50 mM ascorbic acid (Sigma-Aldrich) was used for the osteogenic differentiation experiments.

The activity of ALP was measured using an ALP assay kit (Jiancheng, China) at day 3, 7 and 14 of osteogenic induction. At each time point, medium was discarded and all cell substrates were rinsed with PBS and lysed with 1% Triton X-100 solution at room temperature for 30 min. Afterwards, the lysates obtained were taken to another 96-well plate and measured according to the kit instruction.

Mineralization was estimated by quantifying the formation of calcium phosphate using Alizarin Red S (ARS) staining (cyagen, China). At day 21, all cell substrates were fixed in 4% paraformaldehyde for 1 h and washed three times with PBS, followed by staining with Alizarin Red solution for 15 min; the cell substrates were subsequently rinsed with sufficient deionized water. After the images obtained using a stereomicroscope, 100 mM cetylpyridinium chloride (Sigma-Aldrich, USA) was added into all cell substrates at room 30 min. At last, the supernatant was measured at 562 nm using a spectrophotometer.

### 2.9 Statistical analysis

The data were expressed as the mean ± standard deviation (SD). All data were analyzed with SPSS software (version 21.0) and statistical analyses were evaluated using the *t*-test. Differences *p* < 0.05 was considered statistically significant.

## 3. Results and discussion

### 3.1 Preparation of 3D HA/PAN composite scaffolds

**Fig. 2:**
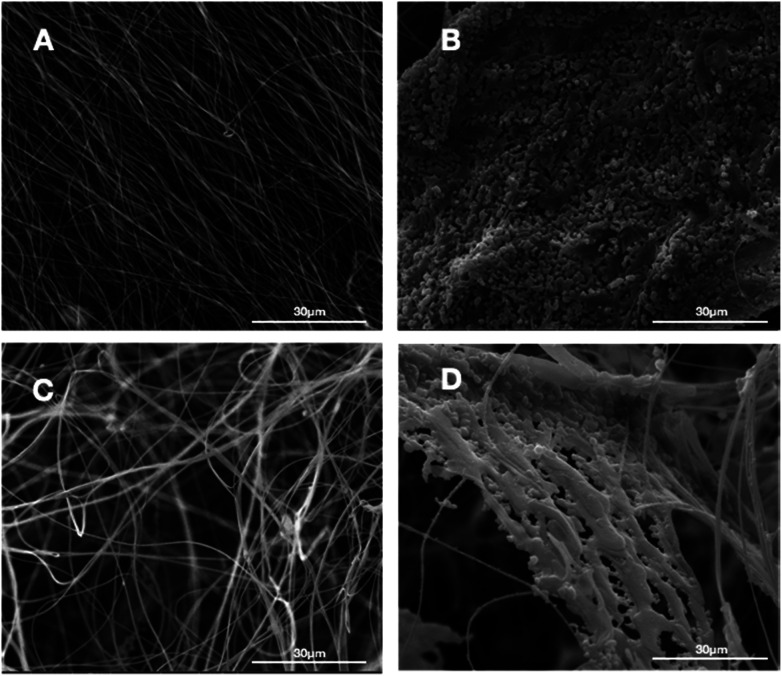
SEM images of PAN membrane and fluffy PAN scaffold before and after mineralization of HA: (A) 2D PAN membrane, (B) 2D HA/PAN composite membrane, (C) 3D PAN scaffold, and (D) 3D HA/PAN composite scaffold.

In this study, we successfully fabricated 3D fluffy HA/PAN composite scaffolds by electrospinning and bio-mineralization. First, we prepared 3D nanofibers as our previous method. Fig. 2C shows the morphology of the prepared PAN fibers in the fluffy PAN scaffolds. Unlike the traditional electrospun PAN fibrous membranes (Fig. 2A), most of PAN fibers in fluffy PAN scaffolds were separated from one another. It was the special structure that made the fluffy PAN scaffolds very soft, which felt like a ball of cotton. And then we took the fluffy PAN scaffolds as template to fabricate the 3D HA/PAN composite scaffolds by bio-mineralization. After 28 days incubation in SBF, the HA coating formed on the surface of PAN fibers which shown in Fig. 2D. The majority of HA fibers maintained in the discrete state while a few fibers intertwined with each other because of the growth of HA coating, which showed similar morphology to fluffy-PAN scaffolds. Besides, we took the traditional PAN membranes as a control to assess the effect of bio-mineralization of HA coating on the spatial structure of PAN scaffolds. As shown in Fig. 2B, the morphology of 2D HA/PAN composite membranes exhibited that the pores in the original PAN membranes were nearly filled by HA coating after 28 days incubation in SBF, only a few pores could be identified on the surface of the 2D HA/PAN composite membranes. Therefore, there was no doubt that cells could only form a cell monolayer or a cluster on the surface of 2D HA/PAN composite membranes, not to mention the 3D cell culture.

### 3.2 Bio-mineralization process of HA

**Fig. 3:**
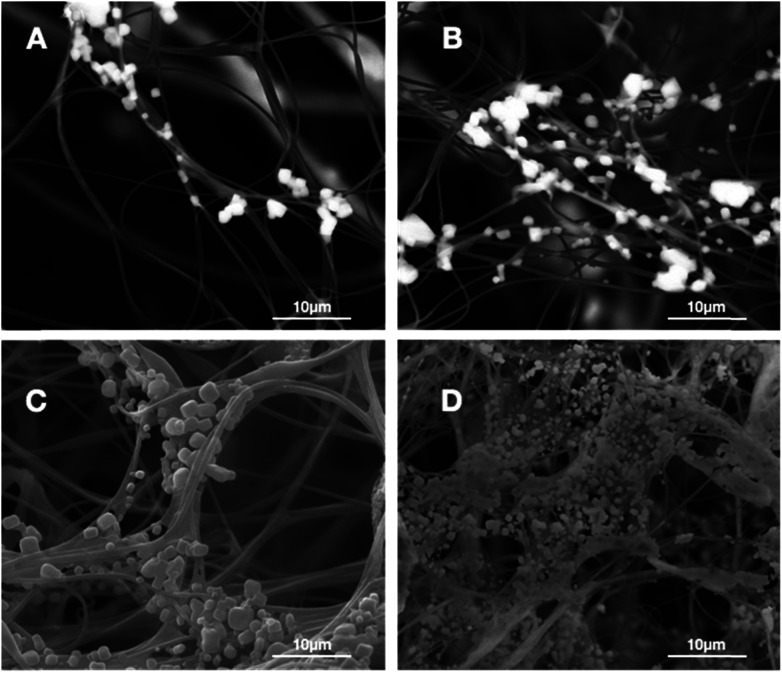
SEM images of the 3D HA/PAN composite scaffold after incubation in SBF at different time points: (A) day 3, (B) day 7, (C) day 14, (D) day 28.

The formation of the HA coating on the surface of PAN fibers in fluffy PAN scaffolds was observed by SEM image as displayed in Fig. 3. A few micro-particles of HA started to appear on the surface of PAN fibers (Fig. 3A) after incubation in SBF for 3 days. Afterwards, a growing deposits of HA particles along the PAN fibers with the growth of incubation time (Fig. 3B and C). Eventually, after 28 days of incubation in SBF, these HA particles grew into a thick and dense HA coating, while the 3D spatial statues of nanofibers were well preserved.

### 3.3 Chemical characterization

**Fig. 4:**
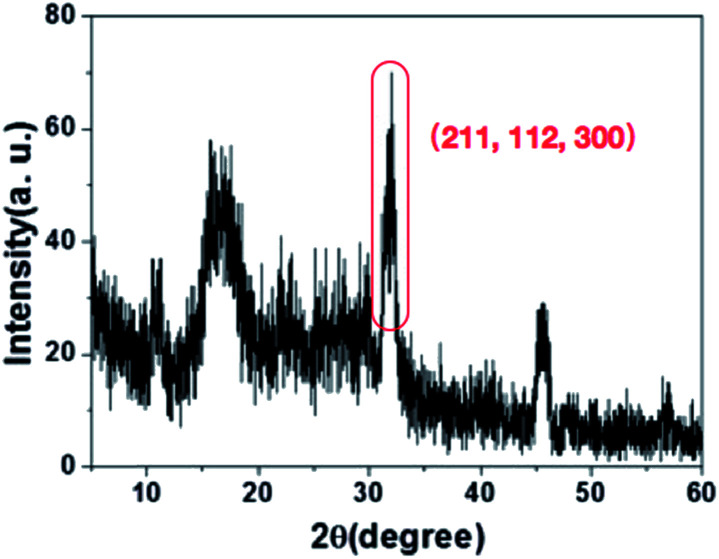
XRD of 3D HA/PAN composite scaffold.

The chemical characteristics of HA on the surface of PAN fibers were observed by XRD. The XRD (Fig. 4) results indicated that the characteristic peaks of HA (211, 112, and 300) were observed after PAN nanofibers coating HA by incubation in SBF.^12^ The XRD spectra combined with SEM images indicated that the successful HA layers were formed on the PAN surface using biomineralizaiton.

### 3.4 Cell cytotoxicity and proliferation

**Fig. 5:**
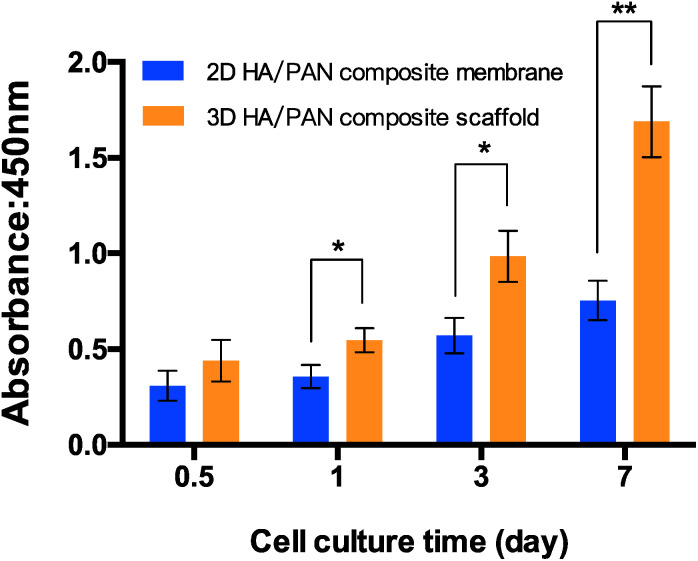
The cytotoxicity and proliferation of BMSCs cultured on 2D HA/PAN composite membrane and 3D HA/PAN composite scaffold at various time points by CCK-8 assay; data are mean ± SD, *n* = 4, *P < 0.05, **P < 0.01.

To assess the effect of the fluffy spatial structure of 3D HA/PAN composite scaffolds for the cell culture, CCK-8 assay was applied to evaluated cell cytotoxicity and proliferation. 2D HA/PAN composite membranes were set as a control. BMSCs were cultured on scaffolds and membranes for a period of 7 days. As shown in Fig. 5, there is a similar cell attachment after 12 h of culture. However, during the culture time of 1 d, 3 d and 7 d, cell proliferation of BMSCs in 3D HA/PAN composite scaffolds is significantly higher than that on the 2D HA/PAN composite membranes. Compared to traditional HA membranes, these results clearly proved that the fluffy spatial structure with high and deep interconnected pores of 3D HA/PAN composite scaffolds could improve cell proliferation.

### 3.5 Cell fluorescence detection and morphology

**Fig. 6:**
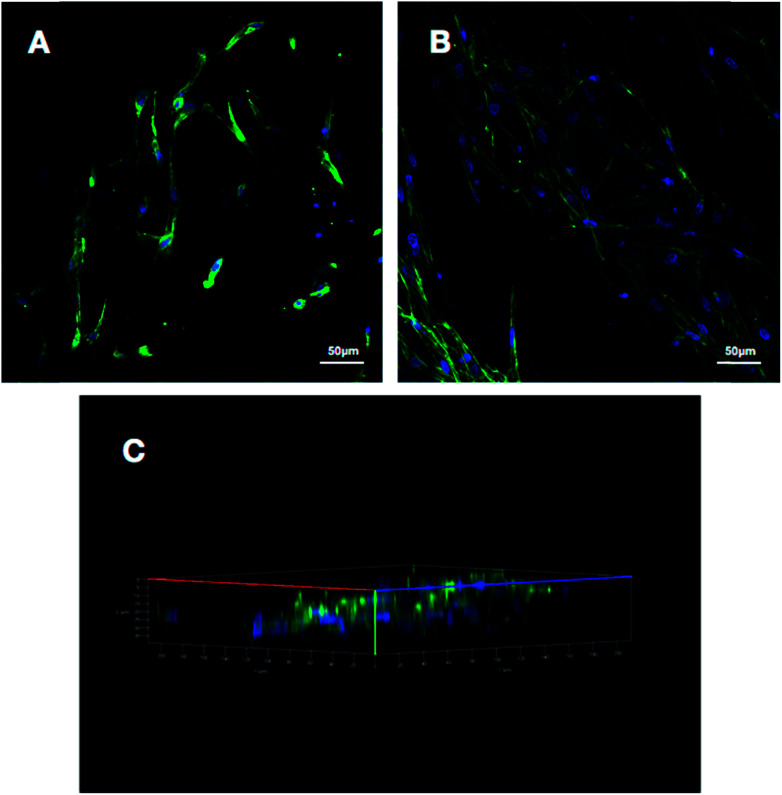
Fluorescence images of BMSCs cultured on different scaffolds at day 3: (A) 2D HA/PAN composite membrane, (B) 3D HA/PAN composite scaffold and (C) three-dimensional reconstruction of 3D HA/PAN composite scaffold cells.

To make an assessment about the biocompatibility and cell affinity of the prepared 3D HA/PAN composite scaffolds and 2D HA/PAN composite membranes, the BMSCs were stained with DAPI and Actin-Tracker Green after 3 days of culture. As shown in Fig. 6B, many cells densely distributed in 3D HA/PAN composite scaffolds, with spindle-like or polygon shape. Besides, it is worth noting that the cells were connected to each other by pseudopodium. Moreover, three-dimensional reconstruction image (Fig. 6C) of the BMSCs in 3D HA/PAN composite scaffolds shows that the cells distributed in multiple levels, which demonstrated the fluffy spatial fibrous structure could allow facile entry of cells into the scaffolds to achieve 3D cell culture. On the contrary, the BMSCs cultured on 2D HA membrane exhibited a bad behavior such as sparse distribution in long fusiform shape and lack of cell–cell contact (Fig. 6A).

**Fig. 7:**
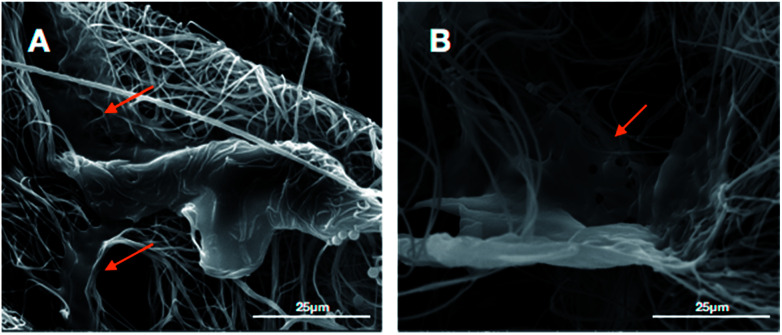
SEM images of BMSCs cultured on different scaffolds at day 3: (A) 2D HA/PAN composite membrane and (B) 3D HA/PAN composite scaffold; yellow arrow: BMSCs.

The micro-morphology of BMSCs cultured on the 3D HA/PAN composite scaffolds and 2D HA/PAN composite membranes were detected by SEM after 3 days of culture. Fig. 7B displays that BMSCs in the 3D HA/PAN composite scaffolds spread widely and formed integrated cell-fiber construct, which was contributed to the maintenance of cell activity. HA on the fibers is disappeared maybe due to the SEM sample preparation process. On the other hand, BMSCs cultured on 2D HA/PAN composite membrane shows a long fusiform shape (Fig. 7A), which was in line with the results of the fluorescence image. These facts strongly supported that the 3D fluffy spatial structure made sure adhesion and migration of BMSCs into the interior of the 3D HA/PAN composite scaffolds easily to promote the formation of 3D cell culture micro-environment, significantly enhanced the cell viability.

### 3.6 *In vitro* osteogenesis study

**Fig. 8:**
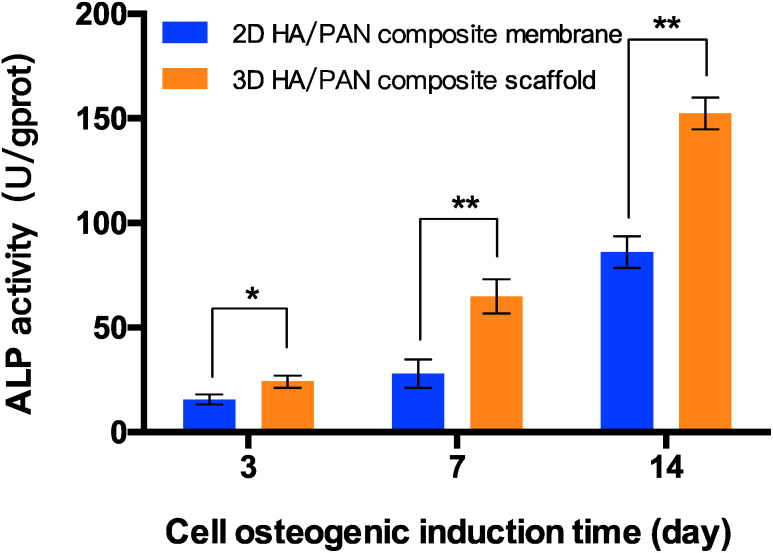
ALP activity of BMSCs cultured on 2D HA/PAN composite membrane and 3D HA/PAN composite scaffold; data are mean ± SD, *n* = 4, *P < 0.05, **P < 0.01.

The capacity of 3D HA/PAN composite scaffolds to improve osteogenic differentiation was estimated. The ALP activity of the BMSCs cultured on scaffolds were investigated, and the result is presented in Fig. 8. BMSCs in the 3D HA/PAN composite scaffolds exhibited a significantly higher ALP activity compared to that on the 2D HA/PAN composite membranes after 3, 7 and 14 days of incubation, which implies that there was a promotion of osteogenic differentiation for BMSCs in 3D HA/PAN composite scaffolds.

**Fig. 9:**
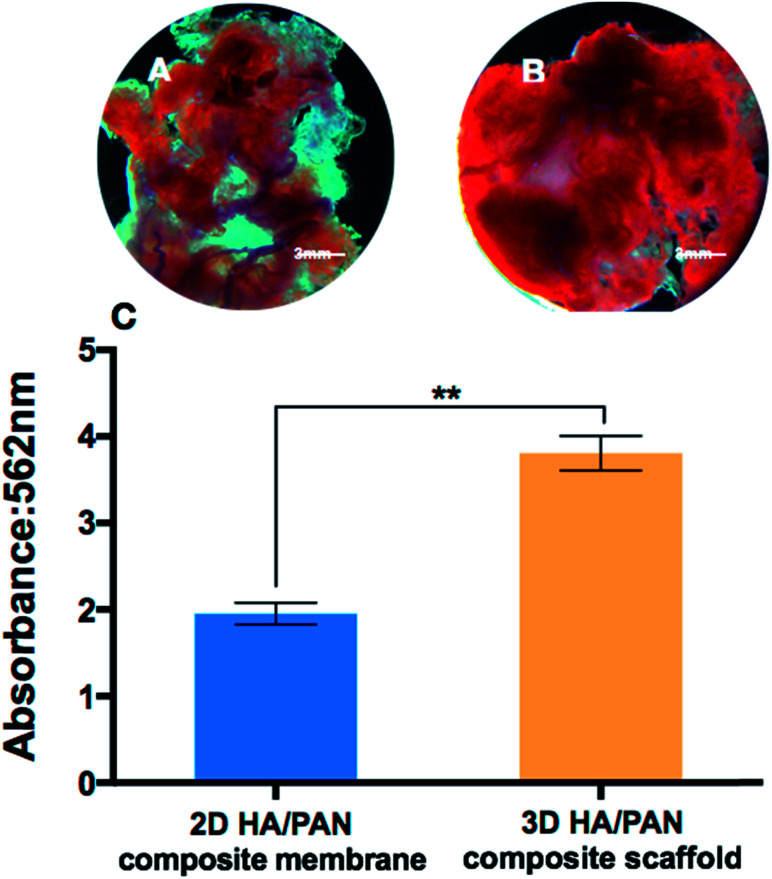
Alizarin red S staining of BMSCs cultured on 2D HA/PAN composite membrane (A) and 3D HA/PAN composite scaffold (B); semi-quantitatively analysis of calcium content (C); data are mean ± SD, *n* = 4, **P < 0.01.

To further study the effects of the 3D HA/PAN composite scaffolds on osteogenic differentiation, mineralization of BMSCs was observed by means of alizarin red S staining. As appeared in Fig. 9, after 21 days of culture, BMSCs in 3D HA/PAN composite scaffolds (Fig. 9B) showed more obvious positive staining of alizarin red S than that on 2D HA/PAN composite membranes (Fig. 9A). Moreover, the result of semi-quantitatively analysis of calcium content (Fig. 9C) was in accordance with the staining result mentioned above, the results demonstrated that there was a remarkably difference between BMSCs cultured in the 3D HA/PAN composite scaffolds and on the 2D HA/PAN composite membranes. These results indicated that the 3D HA/PAN composite scaffolds facilitated the osteogenesis differentiation of BMSCs compared to the 2D HA/PAN composite membranes.

All results suggested that the novel 3D HA/PAN composite scaffolds played a significant role in cell proliferation and differentiation, which indicated a biomimetic micro-environment could regulate the osteogenic behaviors of BMSCs. Moreover, the fluffy scaffold can be tailored to different shapes and sizes meeting the demands in practical application. Thus, we strongly believed that the 3D HA/PAN composite scaffolds could be potential candidates in bone repairing and regeneration in the future. Meanwhile, co-seeding BMSCs and endothelial cells, or carrying some growth factors like SDF-1 and VEGF to promote angiogenesis needed to be further studied, because vascularized is the vital factor in the process of bone regeneration.

## 4. Conclusions

In summary, we developed a novel fluffy HA based fibrous scaffold successfully by an optimized electrospinning technique incorporating with a bio-mineralization process. The 3D HA/PAN composite scaffolds possessed fluffy spatial fibrous structure. The individual structure guaranteed the BMSCs entering the interior of 3D HA/PAN composite scaffolds and achieved 3D cell culture. More importantly, compared to the 2D HA/PAN composite membranes, the 3D HA/PAN composite scaffolds remarkably improved cell adhesion, proliferation, osteogenic differentiation and mineralization of BMSCs. Thus, we expected that the 3D HA/PAN composite scaffolds would have a potential application in the field of bone tissue engineering.

## Conflicts of interest

There are no conflicts to declare.

## Supplementary Material

RA-008-C7RA12449J-s001
