# Supplementary material for: A three-dimensional hydroxyapatite/polyacrylonitrile composite scaffold designed for bone tissue engineering
Source: RSC Adv. 2018 Jan 8;8(4):1730–6. doi: 10.1039/c7ra12449j (PMC9077050; doi:10.1039/c7ra12449j)
Supplement: RA-008-C7RA12449J-s001 [file RA-008-C7RA12449J-s001.pdf]

Electronic Supplementary Material (ESI) for RSC Advances.
This journal is © The Royal Society of Chemistry 2017

A three-dimensional hydroxyapatite/ polyacrylonitrile composite scaffold designed for bone tissue engineering

Shuyi Wu,[#a] *Jieda Wang,*[#a] *Leiyan Zou,*[a] *Lin Jin,*[*b,c] *Zhenling Wang,*[*b] *Yan Li*[*a]

[a]Department of Prosthodontics, Guanghua School of Stomatology, Hospital of Stomatology, Sun Yat-sen University; Guangdong Provincial Key Laboratory of Stomatology, No. 56 Lingyuan Road, Guangzhou, 510055, P. R. China

[b] Henan Key Laboratory of Rare Earth Functional Materials, Zhoukou Normal University, 466001, P. R. China

[c]International Joint Research Laboratory for Biomedical Nanomaterials of Henan, Zhoukou Normal University, Zhoukou, 466001, P.R. China

In this study, 3D nanofibers were prepared using three steps. First, we prepared 3D nanofibers through an improved electrospinning technique (Fig S 1 and Fig. 2b). First, polyacrylonitrile (PAN, MW=100k) was dissolved in the solvent of N, N-dimethylformamide (DMF) (W: V=10%). Second, the mixed solution was fed into a syringe capped with a 0.22 gauge blunt-tipped needle nozzle and driven by a syringe pump (Silugao CO., Beijing, China) at 1.0 mL/h voltage of 15 kV was supplied by a high DC power supply (Dongwen High Voltage, China) between the tip of the needle and the collector at a distance of 10 cm. Then the PAN fibers were spun into a borosilicate beaker (1000mL) with 800mL of ethanol, and then the nanofiber dispersion was replaced by 800mL DI water three times with rigorous shaking. Finally, homogenization of nanofibers was prepared, freeze-drying assembly and the freeze-drying process.[S1]

For 2D nanofiber fabrication, The electrospinning was performed using a customized spinning system. The solution of PAN and electrospinning process were treated as 3D nanofibers fabrication. The obtained nanofibers were deposited onto the aluminium foil-covered collector (Fig. S2).

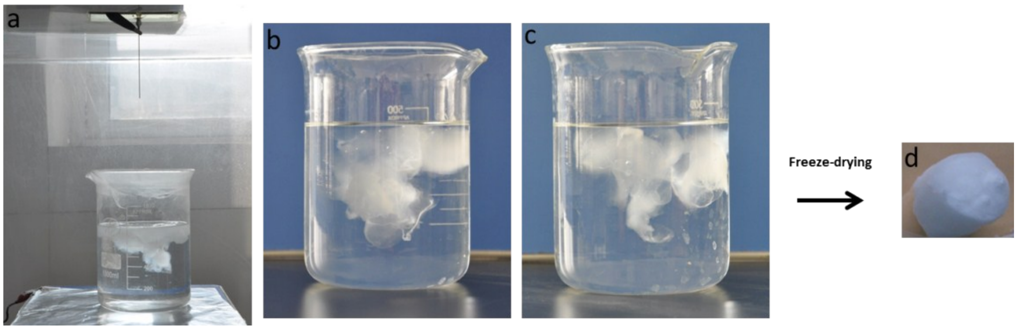

Fig. S1. The fabrication steps of the 3D PAN nanofibers. (a) Electrospinning the PAN nanofibers into ethanol solution. (b) The obtained mixed ethanol nanofiber solution (c) The water nanofiber solution which was prepared by DI water with rigorous shaking. (d) 3D nanofibers are prepared after freeze-drying.

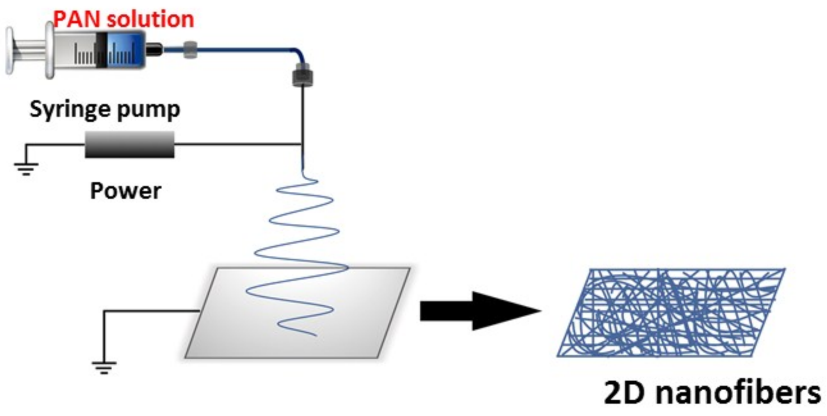

Fig. S2. Schematic diagram of the 2D nanofibers.

[S1] L. Jin, Z.-Q. Feng, T. Wang, Z. Ren, S. Ma, J. Wu, D. Sun, *J. Mater. Chem. B* 2014, **2**, 129-136.
